# Deregulation of neuronal miRNAs induced by amyloid-β or TAU pathology

**DOI:** 10.1186/s13024-018-0285-1

**Published:** 2018-10-12

**Authors:** Annerieke Sierksma, Ashley Lu, Evgenia Salta, Elke Vanden Eynden, Zsuzsanna Callaerts-Vegh, Rudi D’Hooge, David Blum, Luc Buée, Mark Fiers, Bart De Strooper

**Affiliations:** 1 VIB Center for Brain & Disease Research, Leuven, Belgium; 2 Department of Neurosciences, Leuven research Institute for Neuroscience and Disease (LIND), KU Leuven, Leuven, Belgium; 3 Faculty of Psychology and Educational Sciences, Laboratory of Biological Psychology, KU Leuven, Leuven, Belgium; 4 Université Lille, INSERM, CHU Lille, UMR-S 1172, LabEx DISTALZ, Alzheimer & Tauopathies, Lille, France; 5 Dementia Research Institute UK, ION, University College London, London, UK

**Keywords:** Alzheimer’s disease, microRNA, miRNA-seq, In situ hybridization, miR-mimic

## Abstract

**Background:**

Despite diverging levels of amyloid-β (Aβ) and TAU pathology, different mouse models, as well as sporadic AD patients show predictable patterns of episodic memory loss. MicroRNA (miRNA) deregulation is well established in AD brain but it is unclear whether Aβ or TAU pathology drives those alterations and whether miRNA changes contribute to cognitive decline.

**Methods:**

miRNAseq was performed on cognitively intact (4 months) and impaired (10 months) male APPtg (APP^swe^/PS1^L166P^) and TAUtg (THY-Tau22) mice and their wild-type littermates (APPwt and TAUwt). We analyzed the hippocampi of 12 mice per experimental group (*n* = 96 in total), and employed a 2-way linear model to extract differentially expressed miRNAs. Results were confirmed by qPCR in a separate cohort of 4 M and 10 M APPtg and APPwt mice (*n* = 7–9 per group) and in human sporadic AD and non-demented control brain. Fluorescent in situ hybridization identified their cellular expression. Functional annotation of predicted targets was performed using GO enrichment. Behavior of wild-type mice was assessed after intracerebroventricular infusion of miRNA mimics.

**Results:**

Six miRNAs (miR-10a-5p, miR-142a-5p, miR-146a-5p, miR-155-5p, miR-211-5p, miR-455-5p) are commonly upregulated between APPtg and TAUtg mice, and four of these (miR-142a-5p, miR-146a-5p, miR-155-5p and miR-455-5p) are altered in AD patients. All 6 miRNAs are strongly enriched in neurons. Upregulating these miRNAs in wild-type mice is however not causing AD-related cognitive disturbances.

**Conclusion:**

Diverging AD-related neuropathologies induce common disturbances in the expression of neuronal miRNAs. 4 of these miRNAs are also upregulated in AD patients. Therefore these 4 miRNAs (miR-142a-5p, miR-146a-5p, miR-155-5p and miR-455-5p) appear part of a core pathological process in AD patients and APPtg and TAUtg mice. They are however not causing cognitive disturbances in wild-type mice. As some of these miRNA target AD relevant proteins, they may be, in contrast, part of a protective response in AD.

**Electronic supplementary material:**

The online version of this article (10.1186/s13024-018-0285-1) contains supplementary material, which is available to authorized users.

## Background

Declarative memory impairment (i.e. deficits in remembering facts and events) is one of the most prominent and progressive neuropsychological hallmarks of Alzheimer’s disease (AD), yet it remains unknown how the neuropathological processes in AD give rise to these mnemonic disturbances. As genetic mutations in familial AD patients occur only in genes linked to amyloid-beta (Aβ) processing, a central and initiating role for Aβ has been postulated, yet the degree of amyloidosis correlates poorly with the extent of cognitive impairment [1]. Conversely, the spreading of neurofibrillary tangle (NFT) pathology corresponds robustly with clinical AD progression [2], but no pathogenic mutations for AD have been found in the TAU gene (MAPT) up to date.

Sporadic AD patients present with predictable patterns of progressive declarative memory impairment, yet they can exhibit highly divergent neuropathological profiles with different degrees of Aβ plaque load, cerebral amyloid angiopathy, severity of brain atrophy and degree of NFT formation and spread [3, 4]. Similarly, mouse models of AD with amyloidosis profiles or TAU inclusion pathology display overlapping profiles of hippocampus-dependent memory deficits, including progressive declarative memory impairments [4–7]. It is noteworthy that in AD mouse models the memory impairments are claimed to precede or occur independently of neuron loss. These clinical and preclinical observations suggest that despite diverging initiating factors, Aβ and TAU pathologies might converge onto common pathways, ultimately leading to the induction of memory deficits.

Given the vast amount of literature demonstrating altered microRNA (miRNA) expression in AD patients [8, 9] and their abundance in the brain and the synaptic compartment [10, 11], it could be assumed that miRNA deregulation play a central role in AD-related cognitive impairments [12]. Whereas some miRNAs may directly contribute to the formation of AD neuropathology by regulating expression of key AD-related genes, such as APP, BACE1 or MAPT [13], others regulate the synaptic environment, by altering dendritic branching, neurite outgrowth and spine morphology [14–17]. miRNAs can be generated in response to synaptic activity [14, 15, 18, 19] and modulate the maintenance of long-term potentiation [14], which is considered the neurophysiological substrate of memory formation [20]. Obviously, it is also possible that miRNA changes are associated with inflammatory or other disease-related processes and it is impossible to exclude a priori whether alterations in miRNA expression have protective or disease causing effects.

The current study explores whether miRNA disturbances are associated with the progressive memory impairments in AD. miRNAseq on mouse models of amyloidosis (APPtg (APP^swe^/PS1^L166P^) [21]) and TAU pathology (TAUtg (THY-Tau22) [22]) and their wild-type littermates (APPwt and TAUwt), before and after the onset of cognitive impairments, identified six miRNAs involved in neuronal and synaptic dysfunction that were commonly deregulated, yet most strongly in APPtg mice. A similar upregulation was confirmed for four miRNAs, i.e. miR-142a-5p, miR-46a-5p, miR-155-5p and miR-455-5p in AD brain. Despite the clear correlation with disease progression, we could not establish an unequivocal link between changes in miRNA level and cognitive behavior in wild-type mice. Therefore, these miRNA are likely not directly causative to cognitive decline in AD. We discuss alternative possibilities how these miRNA could be involved in AD.

## Methods

### Mice

All animal experiments were conducted according to protocols approved by the local Ethical Committee of Laboratory Animals of the KU Leuven (governmental licence LA1210591, ECD project number P202–2013) following governmental and EU guidelines. APPtg (APP^swe^/PS1^L166P^ [21]) mice express *APP*^*Swe*^ and *PSEN1*^*L166P*^ transgenes under the *Thy1.2* promoter; TAUtg (THY-Tau22 [22]) mice, express the 412 aa isoform of the human 4-repeat *MAPT* gene containing the G272 V and P301S mutation under the *Thy1.2* promoter. All mice have been backcrossed to C57BL/6 J for more than 9 generations. Male mice (transgenic (tg) and wild-type (wt) littermate controls) were sacrificed at 4 months (4 M, average 123.8 days, SD 1.84 days) or 10 months of age (10 M, average 299.8 days, SD 2.22 days), giving rise to 8 experimental groups (*n* = 12 per group): APPwt 4 M, APPwt 10 M, APPtg 4 M, APPtg 10 M, TAUwt 4 M, TAUwt 10 M, TAUtg 4 M, TAUtg 10 M. An independent cohort of 4 M and 10 M APPtg and APPwt littermates (*n* = 7–9/group) was used for qPCR validation. Following cervical dislocation, both left and right hippocampi were microdissected and snap-frozen in liquid nitrogen. Samples were stored at -80 °C.

### Human samples

Hippocampal tissue samples were obtained from the London Neurodegenerative Diseases Brain Bank and collected in accordance to British legislation and their ethical board [23]. The human study was evaluated and approved by the ethical committees of Leuven University and UZ Leuven [23].

### RNA extraction

The left hippocampus was homogenized in TRIzol (Invitrogen, Carlsbad, CA, USA) using 1 ml syringes and 22G/26G needles and purified on mirVana spin columns according to the manufacturer’s instructions (Ambion, Austin, TX, USA). RNA purity (260/280 and 260/230 ratios) and integrity was assessed using Nanodrop ND-1000 (Nanodrop Technologies, Wilmington, DE, USA) and Agilent 2100 Bioanalyzer with High Sensitivity chips (Agilent Technologies, Inc., Santa Clara, CA, USA) and Qubit 3.0 Fluorometer (Life Technologies, Carlsbad, CA, USA), respectively (see Additional file 1: Table S1).

### Library construction, sequencing and mapping

The miRNA library was prepared by the Genomics Core (UZ Leuven) using Illumina TruSeq Small RNA Sample Prep Kit (Illumina, San Diego, CA, USA), adding 2 μl of 50% PEG8000 in the 3′ ligation and with 13 cycles of cDNA amplification. Library construction of 96 samples was performed in 4 different batches of 24 samples, with 1–5 samples per experimental group within each batch. Three libraries were pooled and all RNAs between 5 and 40 bps in length were selected using the BluePippin (Sage Science, Beverly, MA, USA). Equimolar concentrations of 8 pools of 3 samples were loaded per lane and 4 lanes in total were used to sequence 50 bps single-end reads using the Illumina HiSeq 2500 (Illumina, San Diego, CA, USA).

Raw reads were trimmed with Flexbar (version 2.5.35, [24]) and filtered to remove unwanted adaptors, low-quality reads and reads with ambiguous nucleotides. Reads with ≥15 nucleotides were mapped to the mm10/GRCm38 *Mus musculus* assembly using Bowtie2 (version 2.2.1, [25]) allowing 1 mismatch. Stranded miRNA read counts were produced by FeatureCounts from the Subread package (version 1.4.4, [26]) using the miRBase gff3 annotation file (version 20, [27]).

### Data pre-processing and differential expression analysis

We found in 2 of the 12 mice in the APPtg 10 M group a 46% lower expression levels of hsa-APPswe, a 26% lower expression of hsa-Psen1L166p and a 24% reduction in mmu-Thy1 gene, whose promoter drives the expression of the transgenes, compared to the other APPtg 10 M mice. In the absence of an explanation, these two mice were excluded from all further analyses (data not shown).

miRNAs with an average of raw read counts < 5 were discarded, leaving 644 miRNAs for differential expression (DE) analysis. Non-biological variation was removed by the *removeBatchEffect* function from limma package 3.22.7 Bioconductor/R [28], preserving biological variation of genotype, age and their interactions in a generalized linear model for the APPtg and TAUtg dataset combined, as well as separately, while removing technical effects such as batch effect, RNA extraction group effects, RNA concentration effects. DE analysis was conducted using a 2-way interaction model (age, genotype, age*genotype) and performing Benjamini-Hochberg *p*-value adjustment for multiple testing. All miRNAseq data has been submitted to the GEO database under accession number GSE110743.

### Functional assessment of miRNA targets

Predicted miRNA targets were obtained from TargetScan Mouse v7.1. Gene Ontology (GO) category overrepresentation among predicted targets for each upregulated miRNA was assessed using the unranked option in GOrilla [29]. Only GO categories with > 5 predicted targets overlapping were considered for the analysis, using FDR-corrected p-value < 0.05 for significance.

### Reverse transcription and real time PCR

One hundred ng of RNA was used for reverse transcription of miRNAs using the miRCURY LNA™ Universal cDNA synthesis kit II (Exiqon, Denmark). Real time semi-quantitative PCR used ExiLENT Sybr Green master mix kit and LNA PCR primers (Exiqon, Denmark). Cp (crossing points) were determined by using the second derivative method. Fold changes were calculated with the ΔΔCt method (Livak & Schmittgen, 2001) using the geometric mean of mmu-let7g-5p and mmu-miR-23b-3p as normalizer.

### Protein extraction, ELISA, western blotting and correlation analyses

The right hippocampi (*n* = 5–6/group) of our miRNAseq mouse samples were homogenized using a mechanical homogenizer, Fastprep tubes and T-PER Tissue Protein Extraction Reagent (#78510, Thermo Fisher Scientific, Belgium) with phosphatase (P0044 and P5726, Merck, Germany) and cOmplete protease inhibitors (#11836145001, Roche/Merck, Germany). The soluble proteins were separated from the insoluble pool by collecting the supernatant after ultracentrifugation (1 h, 4 °C, 55000 rpm; TLA 100.4 rotor, Beckman Coulter, France). For insoluble Aβ extraction, the pellet was resuspended in 2 volumes (vol: wet weight of tissue) of GuHCl (6 M GuHCl/50 mM Tris-HCl, pH 7.6) with cOmplete protease inhibitors and sonicated for 30s. After 1 h incubation at 25 °C and ultracentrifugation (20 min, 70.000 rpm, 4 °C; TLA 100.4 rotor, Beckman Coulter, France), the supernatant is diluted 12× in GuHCl diluent buffer (20 mM phosphate, 0.4 M NaCl, 2 mM EDTA, 10% Block Ace, 0.2% BSA, 0.0% NaN3, 0.075% CHAPS, pH 7.0) with cOmplete protease inhibitors. Both soluble and insoluble protein fractions were used for detection of human Aβ40 and Aβ42 by standard sandwich ELISA as described before [30].

For detection of total and phosphorylated TAU, the soluble protein fractions were diluted in 1× SDS-PAGE loading buffer with 5% β-mercaptoethanol, boiled (5 min at 96 °C) and briefly centrifuged. Electrophoreses was performed with 7.5 μg (total TAU) or 15 μg (phosphorylated TAU) of protein on NuPAGE Novex 4–12% Bis-Tris gels (Life Technologies, Thermo Fisher Scientific, Belgium), and after transfer, nitrocellulose membranes were blocked for 1 h in blocking solution (5% milk powder in 0.1% TBS-Tween20). Antibody incubation was performed in blocking solution; for the primaries (mouse TAU-5 (total TAU, 1/1000; Ab80579, Abcam, UK); AT8 (1/500; MN1020) and AT270 (1/500; MN1050, Pharmingen, BD Biosciences, Belgium); β-actin (1/100.000; A5441, Sigma-Aldrich, Belgium)) overnight at 4 °C, and for the secondary (goat anti-mouse, 1/10.000; 170–6516, Bio-Rad, USA) for 1 h at room temperature. Blots were developed using chemiluminescence (Perkin Elmer, USA) and normalized to β-actin expression.

Given that not all data was normally distributed, Spearman correlation analysis and False Discovery Rate (FDR) *p*-value adjustment (padj) was performed between the mouse miRNAseq-derived and normalized miRNA expression values, ELISA-based soluble and insoluble human Aβ40 and Aβ42 levels and immunoblotting-based total TAU and phosphorylated TAU levels. The same was done for the human qPCR-based miRNA expression values (this study) and the immunoblotting-based levels of total TAU, AT8, AT270, full length APP, soluble APPβ, and APP C-terminal fragments (CTFs) in the same samples, which were analyzed in a previous study [31].

### In situ hybridization for miRNAs and immunofluorescence

In situ hybridization (ISH) probes (Ribotask and Exiqon, Denmark) against the mature sequence of miRNAs [32–34] were used (Additional file 1: Table S1). Slices (50 μm) of 4% paraformaldehyde perfused mouse brain (APPtg 4 M, APPtg 10 M, APPwt 4 M, APPwt 10 M) were mounted, dried at room temperature (RT), post-fixed with 4% ice-cold paraformaldehyde, heated 3× to boil in the microwave in sodium citrate buffer (10 mM, 0.05% Tween 20, pH 6.0), washed in methylimidazole buffer (2 × 10 min; 0.13 M 1-methylimidazole, 300 mM NaCl, pH 8.0) and fixed with EDC (0.16 M, pH 8.0) in methylimidazole buffer for 1.5 h at 25 °C in a humidified chamber using hybridization covers. After PBS washes, slides were acetylated (1 min, RT) and pre-hybridized (1 h) in hybridization solution (50% formamide, 5xSSC, 1× Denhardt’s solution and 500 μg/ml yeast t-RNA for Ribotask probes). miRNA or scrambled probes (40 nM) were linearized in hybridization buffer (4 min, 94 °C). Sections were hybridized (1.5 h, for temperature see Additional file 1: Table S1). Following stringency washes (at hybridization temperature: 3 × 0.2xSSC and 1 × 0.5xSSC; at RT 1× 2xSSC), slides were incubated with 3% H_2_O_2_ (7 min, RT), and in blocking buffer 1 (1 h, RT; 0.1 M Tris-HCl, 0.15 M NaCl, 0.5% Blocking Reagent (Roche Diagnostics, Belgium) and 0.5% BSA). Subsequently, sections were incubated (1 h, RT) with anti-fluorescein HRP-conjugated antibody (Roche Diagnostics, Belgium) in blocking buffer 1 and developed with TSA Plus Fluorescein reagent (8 min, RT; Perkin Elmer, USA).

Antibodies 6E10 (1/150, Biolegend, USA, #803002), GFAP (1/200, Dako-Agilent, USA, #Z033401) were used for immunofluorescence. Sections were blocked with blocking buffer 2 (1 h, RT; 5% normal goat serum in 0.2% PBS-TritonX 100). Primary antibodies were incubated overnight at 4 °C (6E10) or for 2 h (GFAP) at RT in incubation solution (0.5% goat serum in 0.2% PBS-T), followed by incubation with the secondary antibody (2 h, RT). DAPI counterstain (10 min, RT; 1/5000, Sigma-Aldrich, Belgium, #D9542) was performed and mounting was carried out in mowiol. Images (z-stacks) were acquired using a Nikon A1R Eclipse Ti confocal microscope.

### Intracerebroventricular injections and behavioral experiments

Male mice (C57BL/6 J, 18–26 weeks old, *n* = 9–12 per treatment group) were implanted with a guide cannula in the lateral ventricle (from bregma AP: − 0.1 mm, ML: − 1.0 mm, DV: − 3.0 mm) under isoflurane anesthesia (5% induction, 2–2.5% maintenance). Mice were infused 1× per week with 2 μl of miR-mimic or negative control oligonucleotide (mature sequence, based on *C. elegans* cel-miR-67: UCACAACCUCCUAGAAAGAGUAGA (miRbase ID: MIMAT0000039) Dharmacon, GE Healthcare, Belgium) mixed in a 1:1 ratio with lipofectamine 2000 (Thermo Fischer Scientific, Belgium).

A titration experiment (*n* = 2 per dose) was performed to determine an adequate dose of miR-mimic infusion (Dharmacon, GE Healthcare, Belgium), i.e. not exceeding 100-fold overexpression after 1 week of infusion (see Additional file 2: Figure S1). MiR-mimic treated mice were sacrificed either 1 week or 48 h after infusion via cervical dislocation and the expression levels of the appropriate miRNA were assessed in the right hippocampus using semi-quantitative PCR (see above). The degree of miRNA overexpression in the miR-mimic treated mice was expressed as fold change compared to equimolar or higher concentrations of negative control-treated mice (i.e. 150 pmol miR-mimic against 150 pmol control; 75 & 15 pmol miR-mimic against 75 pmol control; 1.5 to 0.0015 pmol miR-mimic against 1.5 pmol control oligonucleotide).

For the behavioral experiments mice were subjected to either 3wks (individual miRNAs) or 7 wks (miR-mix) of miR-mimic overexpression followed by behavioral assessment (see Fig. 4a), splitting the mice over four separate batches of behavioral experiments: miR-142a (15 pmol) vs negative control (15 pmol); miR-146a (15 pmol) & miR-155 (1 pmol) vs negative control (15 pmol); miR-10a (0.1 pmol), miR-211 (0.1 pmol) & miR-455 (0.15 pmol) vs negative control (0.15 pmol); miR-mix (31.35 pmol) vs negative control (31.35 pmol). Behavioral assessment was performed using the open field, T-maze, context- and cue-dependent fear conditioning, Morris Water Maze (MWM), novel object recognition (NOR) and social preference/social novelty test (SPSN). Mice were sacrificed by cervical dislocation 7 days after the last injection and hippocampi were dissected, snap frozen and stored at − 80 °C, before processing samples for semi-quantitative PCR (see above).

### Locomotion and anxiety-like behavior

After 30 min dark adaptation, the mouse was placed in a brightly lit (465 lx) 50 × 50 cm square arena of transparent plexiglass, 1 min habituation and 10 min recording [35]. Mice were tracked in the arena with ANY-maze^tm^ Video Tracking System software (Stoelting Co., IL, USA), recording total path length (locomotor behavior) and time spent and number of entries in the corners (7 × 7 cm), walls and center circle of the open field (anxiety-like behavior).

### Spatial working memory

The mouse was placed in the start arm of the T-maze and free to enter the left or right arm (familiar arm), after which the other arm (novel arm) was blocked. After 30 min, the mouse was re-introduced into the maze with all arms accessible, and the total path length, number of entries and total time spent in each arm was recorded using ANY-maze^tm^ software.

### Fear conditioning

Mice were habituated to the contextual fear response (CFR) cage (StartFear cage, Panlab, Cornella de Llobregat, Spain) for 5 min. Context- and cue-dependent fear conditioning was induced on day 2 by presenting 2 conditioned stimuli (i.e. 30s tone, 4 kHz, 80 dB, inter-stimulus interval 1 min) which co-terminated with a 2 s 0.3 mA foot shock [36]. Freezing behavior was recorded 24 h later (sensitive Weight Transducer System (Panlab, Cornella de Llobregat, Spain)) as a proxy for memory retention in the conditioned context (no lights, grid floor, ethanol-based odor for 5 min), and in a novel context (lights on, white plastic floor and mint odor, 3 min) and in combination with the auditory cue (3 min).

### Spatial learning

Mice were trained for 2 × 5 days in a 150 cm circular Morris water maze (MWM) pool filled with opaque (non-toxic white paint) water (26 °C) [35], where a 15 cm round platform was submerged in a fixed position 1 cm below water level. Daily training session consisted of 4 trials (15-30 min intervals) starting from 4 randomized starting positions. Mice that failed to locate the platform within 2 min, were gently guided to the platform and left there for 10s. Probe trials were conducted on day 6 and 12 during the 10-day training period, where the platform was removed from the pool and mice swam freely for 100 s. ICV injections were administered after the probe trial. Mice were tracked with EthoVision tracking equipment and software (Noldus, Wageningen, the Netherlands), calculating escape latency, path length and swimming velocity during training, and, during the probe trial, time spent in each quadrant and latency to first enter the target quadrant and target position.

### Recognition memory

In the novel object recognition (NOR), mice were presented for 10 min with 2 randomized identical objects (blue or yellow LEGO® blocks built in different ways, a liquid filled 50 ml falcon tube or a Playmobil® Christmas tree) placed 10 cm from the corners on the diagonal line of the open field arena. After 1 h, the left or right object was replaced by a different object. Exploration lasted 5 min and was scored manually for each object, being defined as directing its nose to the object (< 1 cm distance) and/or touching the object with its nose. Memory performance was calculated by the D2 discrimination index: (time spent exploring novel object – time spent exploring the familiar object)/ total time spent exploring both objects [37].

### Sociability/preference for social novelty test

Sociability and social memory were assessed in the social preference/social novelty test (SPSN), as described in [38]. In short, a test mouse was placed in the middle compartment of a 3-compartment transparent Plexiglas arena (94x28x30 cm), divided by manually guided sliding doors. After 5 min habituation, a stranger mouse was placed in a cylindrical cup in the left or right compartment, and the test mouse could explore all 3 compartments for 10 min during this sociability trial. Subsequently, during the social novelty test a second stranger mouse was placed in a cylindrical cup in the free compartment (left or right) and the test mouse could again explore all 3 compartments for 10 min. Using nose tracking and a virtual 5 cm perimeter around the cylindrical cups holding the stranger mouse, social exploration and distance travelled was assessed using ANY-maze^tm^ software.

## Results

Hippocampi from APPwt, APPtg, TAUwt and TAUtg mice were harvested at 4 months (4 M) and 10 months (10 M) of age, yielding 8 experimental groups. At 4 M APPtg mice show modest levels of soluble and insoluble Aβ40 and Aβ42, which drastically exacerbates at 10 M of age (see Additional file 3: Fig. S2A), which is in line with earlier reports [21]. Although TAU tangle-like pathology does not occur until 12 M in TAUtg mice, levels of total TAU and phosphorylated TAU are similarly increased in 4 M and 10 M mice compared to TAUwt mice, as measured by AT8 and AT270 (see Additional file 3: Figure S2C-E). Both mouse models do not demonstrate hippocampal deficits at 4 M [5, 21, 22], yet display very similar hippocampus-dependent memory deficits at 10 M [5].

**Fig. 1:**
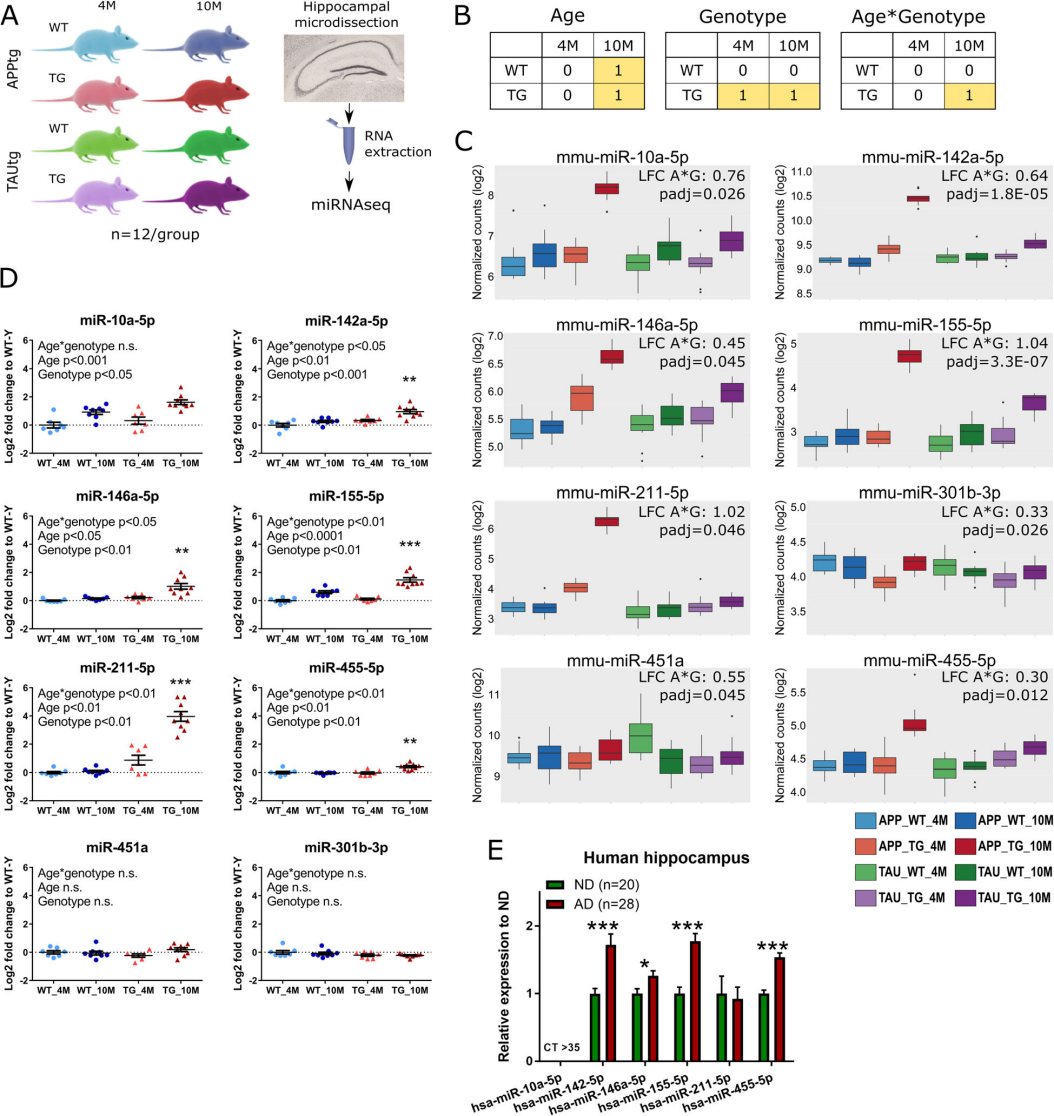
Deregulated miRNAs in hippocampus of APPtg and TAUtg mice and human AD patients. **a**) Experimental design for sequencing using *n* = 12 per experimental group. **b**) Explanation of the 2 × 2 linear model, where those cells labeled with 1 are compared to the cells labeled with 0. In the age comparison, miRNA expression in all 10 month old (M) mice is compared to all 4 M mice. In the genotype comparison, miRNA expression in all transgenic (TG) mice is compared to all wild-type (WT) mice. In the age*genotype comparison, we assess which miRNAs are differentially expressed in the 10 M TG mice compared to all other groups. **c**) The 8 selected miRNAs that became significantly deregulated (FDR-corrected *p*-value< 0.05) in the miRNAseq experiment with increasing pathology and cognitive impairment (age*genotype effect) in APPtg and TAUtg mice combined with more than 20% change. **d**) Validation of 8 deregulated miRNAs in a new cohort of APPtg and APPwt mice at 4 M and 10 M using qPCR (*n* = 7–9/group), expressed relatively to the 4 M WT mice. Significant age*genotype interaction effects were found for miR-142a-, miR-146a-, miR-155-, miR-211- and miR-455-5p, with the 10M TG mice being significantly different from the other 3 groups in Tukey’s post hoc test (**, *p* < 0.01; ***, *p* < 0.001). For miR-10a, significant main effects for age and genotype were found, but no significant age*genotype interaction. miR-451a and miR-301b-3p remained unchanged. **e**) qPCR expression of the 6 validated miRNAs in hippocampus of AD patients and non-demented (ND) controls, demonstrating significant differences for hsa-miR-142-, −miR-146a-, −miR-155- and -miR-455-5p, whereas hsa-miR-10a-5p was too lowly expressed for qPCR assessment (t-test with Benjamini-Hochberg p-value adjustment; *, *p* < 0.05; ***, p < 0.001)

By using http://scotty.genetics.utah.edu [39] and human AD brain miRNAseq data [40] as pilot data, we calculated that *n* = 12/group and 7 million reads/sample would enable us to detect 55% of miRNAs with a significant (*p* < 0.05) 1.3 fold change and > 75% of miRNAs with 1.5 fold change (p < 0.05). miRNAseq was therefore performed on 96 hippocampal samples (see Fig. 1a for a schematic representation of the workflow), yielding on average 7.7 million raw reads per sample. Raw reads were mapped back to 644 miRNAs, discarding lowly expressed miRNAs (≤ 5 raw counts across 10 samples).

We studied alterations in miRNA expression patterns by using a 2 × 2 linear model (see Fig. 1b). Through this model we can assess the effects of age, genotype and the age*genotype interaction. The age comparison identifies which miRNAs are differentially expressed between 4 M and 10 M old mice, irrespective of the genotype. In the genotype comparison, we identify which miRNAs are different between WT and TG mice, irrespective of age. In the age*genotype interaction comparison we assess which miRNAs are changed with aging but that are unique to the TG mice only. The latter thus provides insight on miRNA alterations that correlate with the establishment of cognitive impairments and the progression of AD pathology in TG mice.

### miRNAs are progressively deregulated in the age*genotype interaction

Overall, the significant (adjusted *p*-value after false discovery rate correction (padj) < 0.05) observed changes in miRNA expression are modest (log2-fold change (LFC): − 0.95 - + 2.23), similar to what we reported in AD patients [40]. Combining the APPtg and TAUtg dataset during differential expression analysis highlights miRNAs that are commonly deregulated. Eight miRNAs are significantly up-regulated with more than 20% change (LFC > 0.26) in the age*genotype interaction when both APPtg and TAUtg mice are taken together, although the pattern of deregulation of miR-451a and miR-301b-3p is less outspoken compared to the other six (see Fig. 1c). miR-10a-5p (LFC: + 0.76), miR-155-5p (LFC: + 1.04) and miR-211-5p (LFC: + 1.02) show the highest up-regulation (see Fig. 1c and Additional file 1: Table S2). Nine out of these 13 miRNA were previously found up-regulated in post mortem AD brains compared to controls [9, 40–46]. Remarkably, no significantly down-regulated miRNAs are observed in the analysis of the two genotypes combined.

The strongest effect appears in APPtg mice in the age*genotype interaction resulting in 18 miRNA that are significantly differentially expressed (Additional file 4: Figure S3). Four out of these 18 miRNAs are also significantly differentially expressed due to genotype alone in APPtg mice (miR-211-5p, miR-142a-5p, miR-146a-5p and miR-301b-3p; Fig. 1c). This indicates that their expression is already profoundly affected at 4 M of age and their deregulation exacerbates at 10 M in APPtg.

Although 6 out of the 8 miRNA derived from the combined genotype*age interaction (see Fig. 1c) show a similar trend in the TAUtg-specific analysis compared to the APPtg-specific analysis (Fig. 1c and Additional file 1: Table S2), the overall deregulation of miRNAs is rather subtle in the TAUtg model, with no miRNAs reaching significance in the age*genotype comparison.

### A stronger genotype effect in TAUtg mice

When comparing the effect of transgene expression alone in the combined APPtg and TAUtg data set, 23 miRNAs are affected in a statistically significant manner (padj < 0.05, see Additional file 4: Figure S3), but effect sizes are modest with maximal changes for miR-146a-5p (LFC: + 0.36) and miR-147-3p (LFC: + 0.34), or miR-451a (LFC: − 0.40) and miR-301b-3p (LFC: − 0.27). By assessing the transgene effects in APPtg and TAUtg mice separately, it becomes clear that TAUtg mice have more significantly differentially expressed miRNAs than the APPtg mice (24 vs 6 miRNAs; padj< 0.05), although the effects are weak. Not only do TAUtg mice display more down-regulated miRNAs (*n* = 19, LFC: -0.74 to − 0.14, for instance miR-487b-5p and miR-451a) than APPtg mice (*n* = 1, miR-301b-3p, LFC: − 0.32), their LFCs are also more pronounced. Conversely, TAUtg and APPtg mice both show 5 significantly up-regulated miRNAs (APPtg, LFC: + 0.23 to + 0.63, including miR-211-5p and miR-146a-5p; TAUtg, LFC: + 0.14 to + 0.31, including miR-7b-5p and miR-344d-3p), although the extent of up-regulation is more pronounced in APPtg mice, and there is no overlap in affected miRNAs.

### Aging-induced miRNA changes

Assessment of the effects of aging (comparing 4 M to 10 M mice) in APPtg and TAUtg mice combined, results in 50 significantly differentially expressed miRNAs (padj < 0.05; see Additional file 4: Figure S3). The most strongly up-regulated “age associated” miRNAs include miR-193a-3p (LFC: + 0.25) and miR-16-1-3p (LFC: + 0.22) and the most strongly down-regulated miRNAs include miR-298-3p (LFC: − 0.92), miR-296-5p (LFC: − 0.78) and miR-296-3p (LFC: − 0.65).

When assessing APPtg and TAUtg mice separately, aging induces slightly more significantly differentially expressed miRNAs in TAUtg than in APPtg mice (30 vs 23, padj< 0.05). Aging is expected to produce similar miRNA changes among the two different transgenes, and we find 8 down-regulated miRNAs (including miR-298-3p (APPtg LFC: − 0.95; TAUtg LFC: − 0.90) and miR-296-5p (APPtg LFC: − 0.76; TAUtg LFC: − 0.80)) and 1 miRNA up-regulated (miR-210-3p (APPtg LFC: 0.14; TAUtg LFC: 0.23)) in both models. Overall, aging induces moderate changes in miRNA expression that are similar among APPtg and TAUtg mice.

To summarize, we can conclude that APPtg and TAUtg transgenes induce transgene-specific and transgene-indistinct changes in miRNA networks. Transgene-specific alterations can occur due to the distinct nature of the mouse models’ pathology, and it is therefore highly intriguing to find overlaps. These overlaps may contribute to shared features in the phenotypes of both mouse models, for instance the shared cognitive phenotype as previously described [5, 21, 22]. We focus our study further on the 8 miRNAs discussed above that show a significant age*genotype effect (padj< 0.05) when combining the APPtg and TAUtg datasets (i.e. commonly deregulated) and show more than 20% change (i.e. LFC > + 0.26 or LFC < − 0.32; see Fig. 1c).

### Confirmation of changes in a separate cohort of APPtg mice and in AD patients

It is important to corroborate changes in miRNA expression by independent methods [40]. We therefore measure expression of the eight selected miRNAs in an independent new cohort of APPtg mice using semi-quantitative qPCR. The changes in miR-301b-3p and miR-451a, in APPtg mice as mentioned above were small and could not be confirmed using qPCR in the new APPtg cohort (Fig. 1d). However, the age*genotype interaction effect for miR-142a-5p, miR-146a-5p, miR-155-5p, miR-211-5p and miR-455-5p (*p* < 0.01; Fig. 1d) is confirmed in the new cohort. miR-10a-5p shows significant main effects for age and genotype, but not for the age*genotype interaction. Thus, the qPCR confirms that miR-10a-5p, miR-142a-5p, miR-146a-5p, miR-155-5p, miR-211-5p and miR-455-5p are truly upregulated in 10 M APPtg mice.

We next analyze the expression of the human homologues of the 6 verified miRNAs in hippocampal material from 28 neuropathologically verified AD patients and 20 non-demented control cases, previously used in [31]. Semi-quantitative qPCR data indicates increased miRNA expression in AD cases of hsa-miR142-5p (+ 72%), hsa-miR-146a-5p (+ 26%), hsa-miR-155-5p (+ 78%) and hsa-miR-455-5p (+ 54%; see Fig. 1e). Hsa-miR-211-5p shows too much variability across the samples, and therefore its alteration in human patients cannot be confirmed. Hsa-miR-10a-5p is too lowly expressed in human hippocampal tissue to be detected (qPCR cycle threshold > 35). We can conclude that miR-142a-5p, miR-146a-5p, miR-155-5p and miR-455-5p are upregulated in both mouse models of neurodegeneration (albeit more strongly in APPtg mice), in an independent cohort of APPtg mice, and in AD patients, whereas the increase in miR-10a-5p and miR-211-5p expression is only found in the APPtg mouse model for AD.

We further assessed whether the increase in miRNA expression was correlated to levels of pathology in both the mouse models of neurodegeneration and in sporadic AD patients. Spearman correlation and FDR *p*-value adjustment was performed on the miRNAseq-based expression of the 6 miRNAs and levels of soluble and insoluble Aβ40 and Aβ42 in APPtg mice and the levels of total TAU and phosphorylated TAU (AT8 and AT270 staining) in TAUtg mice. High (0.97<*R* > 0.58) and significant (padj< 0.005) correlations are found between all Aβ species and the miRNAs of interest in the amyloidosis mouse model (see Additional file 3: Figure S2B. By contrast, significant correlations could only be found between total TAU and miR-155-5p and miR-455-5p and between TAU Thr181 phosphorylation (AT270) and miR-146a-5p and miR-155-5p in the tauopathy mice (see Additional file 3: Figure S2E). In human samples, correlating protein levels of TAU, AT8, AT270, full length APP, soluble APPα and APP c-terminal fragments, as measured in [31], with the qPCR-based expression levels of the 5 expressed miRNAs, demonstrated that miR-155-5p is also significantly and positively correlated (*R* = 0.51, padj< 0.01) with levels of TAU Thr181 phosphorylation (AT270; see Additional file 3: Figure S2F).

### Deregulated miRNAs are predominantly expressed in neurons and can regulate neuronal functions

**Fig. 2:**
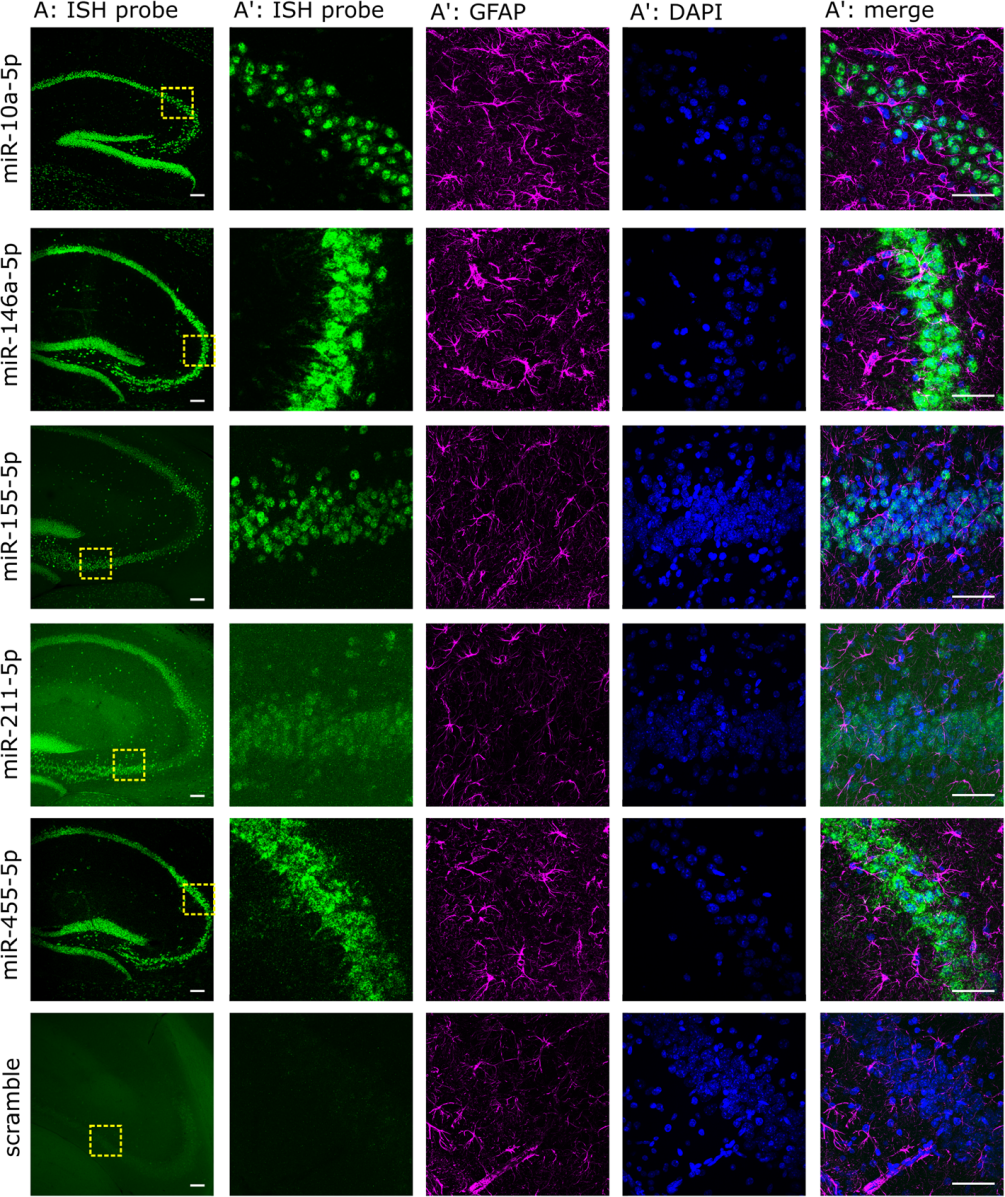
Deregulated miRNAs are expressed in neurons. Fluorescent in situ hybridization of 5 deregulated miRNAs combined with a nuclear counterstain (DAPI) and immunofluorescence for activated astrocytes (GFAP) in 10 M APPtg mice. A) All miRNA probes (rows) demonstrate high expression in hippocampal neuronal layers, except for the scrambled probe. Scale bar, 50 μm. A’) higher magnification of the yellow box indicated in A, demonstrating little expression of miRNA ISH probes in GFAP-positive cells. Scale bar, 100 μm

The two major cellular alterations in the mouse models used here are gliosis and loss of synapses and/or neurons [21, 22]. We therefore used fluorescent in situ hybridization (FISH) to find out in which cells the identified miRNAs are expressed. Five out of six miRNAs (miR-10a-5p, miR-146a-5p, miR-155-5p, miR-211-5p, miR-455-5p) are expressed predominantly in neurons, as shown by the clear binding of miRNA probe within the neuronal layers of the hippocampus (see Fig. 2). Specific miR-142a-5p FISH signal (i.e. different from scrambled probe) is not achieved in our hands, although this miRNA displays a predominant neuronal expression in Simian monkey brain [34]. Very little to no FISH-positive signal is observed in Gfap-positive astrocytes (see Fig. 2) or Iba1-positive microglia for all 5 miRNAs (data not shown). Thus, our data suggests that the 5 miRNAs, and likely miR-142a-5p, are mostly expressed in neurons.

**Fig. 3:**
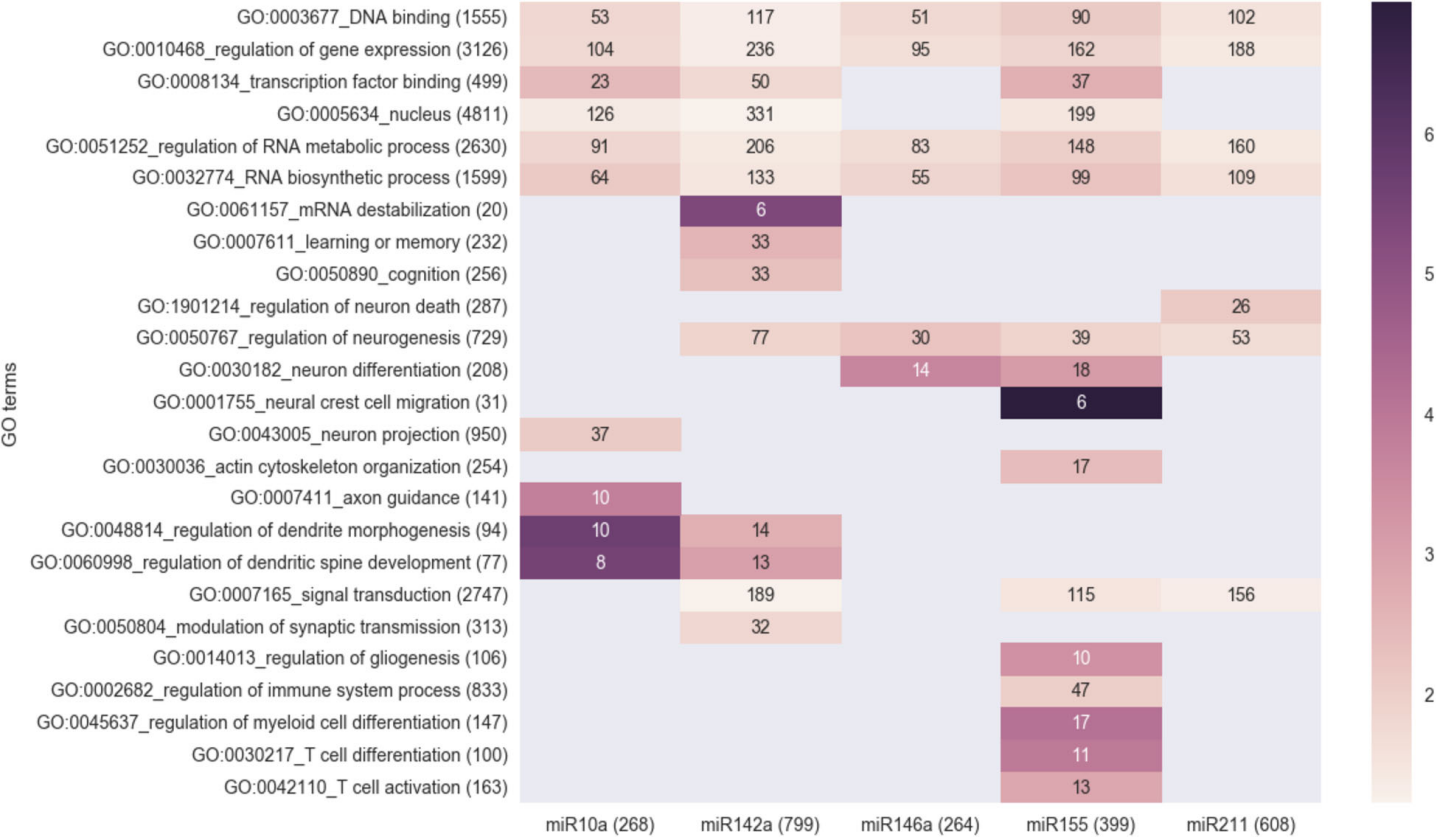
Gene ontology (GO) enrichment analysis on predicted targets of each upregulated miRNA. Numbers between brackets are the number of genes within each GO category or the number of predicted targets for each miRNA. Colors represent the enrichment score and only significantly enriched GO terms/miRNA target combinations are depicted (FDR-corrected *p*-value< 0.05). Numbers within each cell represent the number of predicted targets that fall into each GO category. No significant enrichment is observed for the predicted targets of miR-455-5p, which is therefore excluded from the heatmap

We identified their predicted targets by TargetScan Mouse v7.1, [47] (see Additional file 1: Table S3 for an overview). Predicted targets of 5 miRNAs (miR-455-5p did not show significant enrichment (FDR-adjusted *p*-value < 0.05) of gene ontology (GO) categories), demonstrate significant enrichment for GO categories as ‘Regulation of neurogenesis’ (GO: 0050767), ‘Signal transduction’ (GO: 0007165) and ‘Regulation of dendritic spine development’ (GO: 0060998; see Fig. 3). Targets of miR-155-5p also enrich significantly for immune-related GO categories, among which ‘Regulation of immune system process’ (GO: 0002682), ‘T cell activation’ (GO: 0042110), ‘T cell differentiation’ (GO: 0030217) and ‘Regulation of myeloid cell differentiation’ (GO: 0045637), most probably due to its reported immune-related function in myeloid cells [48]. However, the importance of this for the alterations in our mouse models is unclear given the high expression of this miRNA in neurons (see Fig. 2).

### Overexpression of miRNAs in wild-type mice does not induce cognitive impairments

**Fig. 4:**
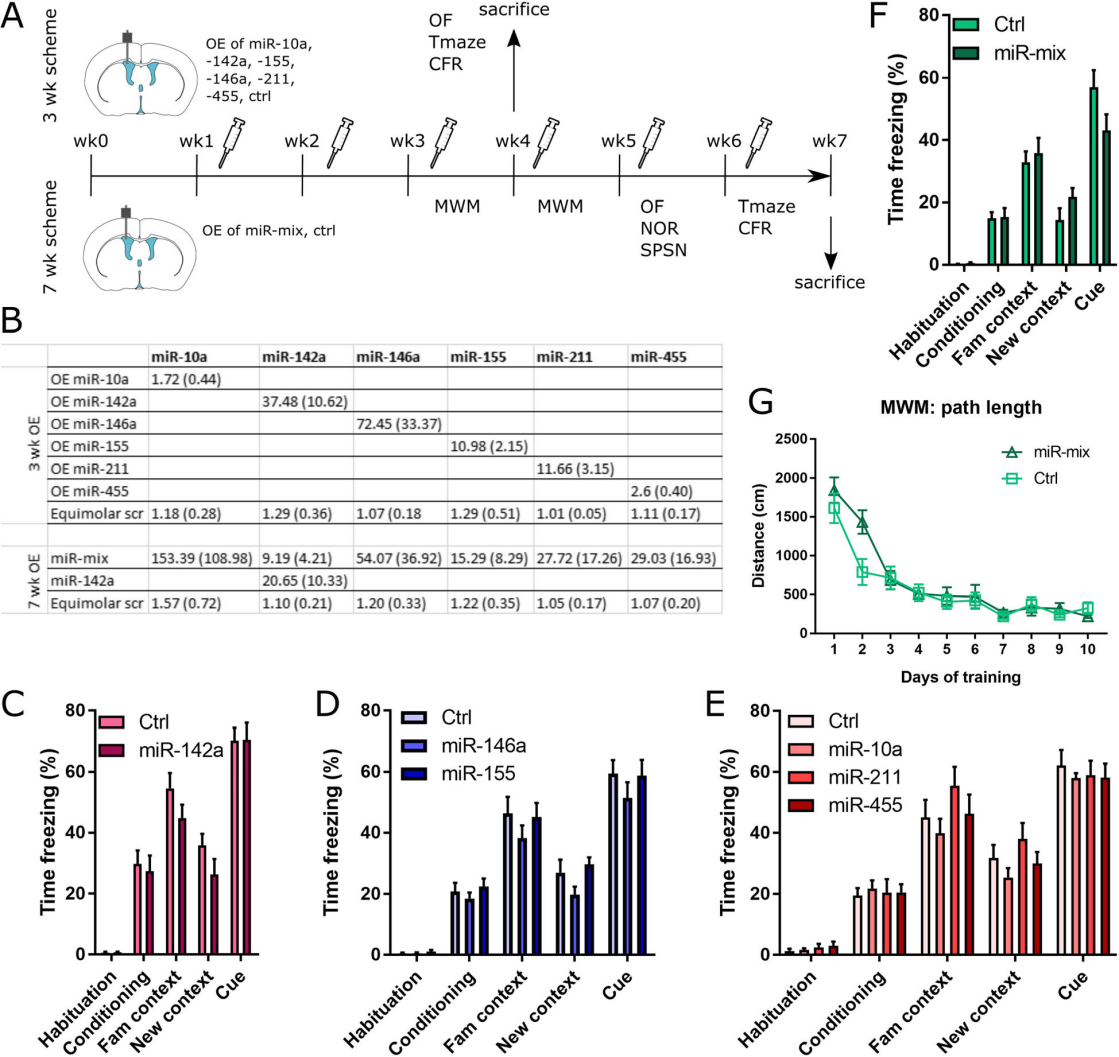
miRNA overexpression does not induce robust cognitive deficits in WT mice. **a**) Outline of behavioral study. Male mice (C57BL/6 J, 18–26 weeks ol, *n* = 9–12 per treatment group) were stereotactically implanted with an intracerebroventricular guide cannula and injected 1× per week with either a miR-mimic to overexpress individual miRNAs (3wk scheme), a mix of 6 miR-mimics (miR-mix; 7wk scheme) or negative control oligonucleotide (Ctrl). **b**) Mean fold change (SEM) compared to equimolar control oligonucleotide of miRNAs in the hippocampus after individual miRNA or miR-mix overexpression. **c**-**e**) Overexpression of individual miRNAs does not induce changes in cued or contextual conditioned fear responses. **f**) Path length in the MWM to find the submerged platform over 10 training days. MiR-mix treatment did not induce significant differences in learning ability. **g**) MiR-mix treatment does not affect cued or contextual conditioned fear response. OE: overexpression; wk.: week; OF: open field test; CFR: conditioned fear response; MWM: Morris water maze; NOR: novel object recognition test; SPSN: social preference/social novelty test

Given that the upregulated miRNAs mainly target neuronal and synaptic transcripts, we wondered whether upregulation of these miRNAs would be sufficient to induce the cognitive alterations observed in APPtg and TAUtg mice [5, 21, 22]. We implanted intracerebroventricular guide cannulas into male C57BL/6 J mice (18–26 weeks of age, *n* = 9–12 per treatment group) and injected weekly a miR-mimic oligonucleotide to overexpress the 6 miRNAs individually or in a mix combining all 6 miR-mimics (miR-mix), or equimolar negative control oligonucleotide, to assess their impact on cognition (see Fig. 4a). Overexpression of one miRNA did not lead to overexpression of any of the other 5 miRNAs (data not shown). The miR-mix treated mice underwent a more extensive cognitive test battery (see Fig. 4a).

Weekly ICV injections of individual miR-mimics or miR-mix leads to a significant increase in the hippocampal expression of each miRNA (see Fig. 4b). miRNA overexpression is well tolerated, with no signs of heightened anxiety or altered locomotor behavior as shown by the open field test (see Additional file 5: Figure S4A, B). However, none of miR-mimic treatments significantly affect cognitive behavior, as measured by the ability to recognize the familiar arm in the T-maze (Additional file 5: Figure S4C), or freezing behavior when presented with the same context or the cued tone that was previously coupled to the shock in the contextual fear conditioning paradigm (see Fig. 4c-e, f). MiR-mix treatment is also not able to induce changes in spatial learning (Fig. 4g), nor in the ability to locate the hidden platform in the Morris Water Maze after 10 days of training (see Additional file 5: Figure S4D). Although both control-treated and miR-mix-treated mice are unable to distinguish the novel stranger from the familiar stranger mouse in the Social Preference/Social Novelty test (see Additional file 5: Figure S4E), their ability to distinguish a familiar and novel object remains intact (see Additional file 5: Figure S4F). Overall, these results indicate that overexpression of the 6 deregulated miRNAs does not induce robust cognitive impairments in C57BL/6 mice.

## Discussion

The current study provides a detailed outlook on the deregulation of the miRNA landscape in APPtg and TAUtg mice during pathology progression and neurodegeneration-related memory impairments and confirms 4 of the 6 identified miRNA changes in human brain. By using an unprecedented number of mice per group (*n* = 12/group) and highly homogeneous groups in terms of sex and age, our statistical power is strong enough to detect even subtle changes in miRNA expression with high confidence.

By performing a side-by-side comparison of APPtg and TAUtg mice we have identified that overexpression of TAU is sufficient to drive miRNA changes from 4 M of age, while miRNA disturbances in APPtg mice mainly occur as amyloid neuropathology has accumulated at 10 M of age. Although TAU can interact directly with RNA and RNA binding proteins [49–51], it remains unclear to date whether TAU plays a direct role in miRNA biogenesis [52] or whether our observed changes in miRNA expression in TAUtg mice are a secondary consequence.

We found 6 miRNAs (miR-10a-5p, miR-142a-5p, miR-146a-5p, miR-155-5p, miR-211-5p and miR-455-5p) to be commonly deregulated between APPtg and TAUtg mice and 4 of these (miR-142a-5p, miR-146a-5p, miR-155-5p and miR-455-5p) were upregulated in AD patients [40, 41, 43, 44, 53]. Upregulation of miR-142a-5p, miR-146a-5p and miR-155-5p has also been found in brains of patients with other neurological illnesses presenting with neuroinflammation and cognitive impairment, including Down’s syndrome, multiple sclerose, amyotrophic lateral sclerosis, virus-induced encephalitis and temporal lobe epilepsy patients [54–62].

It has been suggested that increases in the expression of miR-142a-5p, miR-146a-5p and miR-155-5p in patients and mouse models of neurodegeneration are due to a combination of disease-associated gliosis and glial expression of these miRNAs, although in situ evidence of glial expression of these miRNAs is lacking [55, 59, 63, 64]. Our ISH data, alternatively, suggest these ‘glia-specific’ miRNAs miR-142a-5p, miR-155-5p and miR-146a-5p are in fact mainly expressed in neurons [34, 60, 62, 65], with minimal expression in astrocytes and microglia, making it unlikely that the upregulated miRNA expression in our mice is from glial origin. Given the responsiveness of miR-146a-5p and miR-155-5p miRNAs to inflammatory factors such as NF-κB [66, 67], and NF-κB also being expressed in neurons [68], it could be speculated that the progressive neuroinflammation in these neurodegenerative mouse models induces neuronal expression of these miRNAs. Given the loss of neurons in patients with AD and in our TAUtg mouse model from 12 M onwards, the increased miRNA levels are real and not caused by changes in cell numbers. Single cell miRNA sequencing techniques are not yet readily available, but once those are developed, these will shed additional light on cell-type specific miRNA and target interactions in healthy and diseased conditions.

In the behavioral experiment we aimed to phenocopy the AD-like hippocampus-dependent deficits of our mouse models in WT mice, by overexpressing the 6 deregulated miRNAs either individually or in a mix. Neither treatment regimen induced robust cognitive deficits in WT mice, indicating that the identified miRNAs are not directly responsible for the disease process. It is difficult to interpret negative data, raising questions regarding the frequency and efficiency of miR-mimic delivery and their functional effect. Yet our titration experiment for dose determination demonstrates good dose-response curves and/or decreasing overexpression levels over time for all the miR-mimic treatments (see Additional file 2: Figure S1), demonstrating the technical feasibility of such infusion experiments. Indeed, both overexpression and downregulation studies in WT and TG mice have successfully been used in the past with the same delivery route and compounds to phenocopy or rescue various aspects of AD-related neurodegeneration or behavioral deficits [31, 69–71]. Thus our original hypothesis that these common upregulated microRNA are involved in the converging memory declining phenotype in these mice models, does not hold. Additional studies are now needed to investigate the alternative hypothesis, i.e. whether downregulating the miRNA using anti-miR oligonucleotides, in the AD model mice would aggravate their phenotypes.

It should be noted that the identified miRNAs have among their predicted targets several AD relevant proteins involved in Abeta generation (e.g. Aph1a, Aph1b) or TAU kinases (e.g. Gsk3β, Cdk5, Camk2a), phosphatases (e.g. Pten, Ppp2ca) and protein TAU itself (see Additional file 1: Table S4). Since the levels of (some) miRNAs correlate with increasing pathology load (see Additional file 3: Figure S2B&E), one is tempted to speculate that upregulation of these miRNAs may indeed exert a protective effect, i.e. dampen pathology. Further work is needed to investigate whether even stronger or earlier overexpression of these miRNAs in neurodegeneration mouse models might have a protective effect on the progression of AD-related pathology in these mice.

## Conclusions

The current study identified 6 miRNAs that become upregulated in 2 mouse models of neurodegeneration, 4 of which could be confirmed in AD patients. Upregulation of these miRNAs in WT mice was insufficient to drive cognitive impairment. It is possible that they contribute along with other deregulated (post)transcriptional pathways to cognitive deficits in AD models, however the opposite interpretation that these microRNA modulate pathology load in these mouse models requires further investigation.

## Additional files

Additional file 1: **Table S1.** Overview of miRNA FISH probes. **Table S2.** All significant miRNAs for age/genotype/age*genotype for APPtg and TAUtg combined, APPtg and TAUtg. **Table S3.** Predicted targets by TargetScan Mouse (v.7.1) for the six identifief miRNAs. **Table S4.** Predicted targets of miR-10a-5p, miR-142a-5p, miR-146a-5p, miR-155-5p, miR-211-5p and miR-455-5p that play a role in the production of amyloid-β or TAU (de)phosphorylation. (XLSX 65 kb)

Additional file 2: **Figure S1.** Semi-quantitative qPCR data of the titration study to assess the degree of miRNA expression after various dosages of miR-mimic oligonucleotide (*n* = 2 per dose). For each miRNA the level of overexpression was determined either 1 week after receiving a 150, 75 or 15 pmol dose (green dots), or after 48 h (blue squares) when receiving either a 15 pmol dose (miR-142a-5, miR-146a-5p), or a 1.5–0.0015 pmol dose (all others). As expected, overexpression levels of miR-142a-5p and miR-146a-5p are higher 48 h than 1 week after infusion. Fold changes per dose are expressed in comparison to the expression levels of the miRNA in negative control infused mice (see Methods). (PNG 2150 kb)

Additional file 3: **Figure S2.** Correlation between miRNA expression and pathology load in APPtg and TAUtg mice and AD patients. A) Hippocampal levels of soluble and insoluble Aβ40 and Aβ42 levels were measured by ELISA in APPwt and APPtg mice at 4 M and 10 M of age. 2-way ANOVA demonstrates significant effects for age, genotype and age*genotype for all measured Aβ species, with 10 M APPtg mice having significantly higher expression compared to all other groups. ***:*p* < 0.001 (Tukey’s post-hoc analysis). B) Spearman correlation with false discovery rate (FDR) *p*-value adjustment, demonstrate significant correlations (*p* < 0.05) between all miRNAs and all Aβ species. C) Representative western blot of total TAU and phosphorylated TAU (as measured by AT8 & AT270). D) Quantification of the blots shown in C), demonstrating significant genotype effects for both TAU and phosphorylated TAU. Levels of phosphorylated TAU are normalized to both β-actin as well as to TAU expression levels. E) Spearman correlation with FDR p-value adjustment between the 6 miRNAs of interest and levels of TAU protein and phosphorylated TAU. The correlation coefficient is only stated in the cells of the table if it was statistically significant (*p* < 0.05), otherwise the cell reads ‘0’. F) Spearman correlation with FDR p-value adjustment between the qPCR-based expression levels of the 5 expressed miRNAs in human tissue and the protein levels of TAU, phosphorylated TAU (AT8 & AT270), full length APP (flAPP), soluble APPβ (sAPPbeta) and APP c-terminal fragments (CTFs) as measured by western blotting in [31]. Only cells with significant (p < 0.05) correlations state the correlation coefficient, the others read ‘0’. (PNG 3343 kb)

Additional file 4: **Figure S3.** Significantly differentially expressed miRNAs after statistical assessment of age*genotype interaction (int), age and genotype (gen) in APPtg and TAUtg mice combined (all) and separate. Colors represent log2(fold change) and only statistically significant (padj< 0.05) miRNAs are depicted. (PNG 2826 kb)

Additional file 5: **Figure S4.** Behavioral assessment after intracerebroventricular injections of individual or a mix of 6 miR-mimic oligos (miR-mix), or negative control (Ctrl). A) miRNA overexpression does not induce changes in locomotor behavior as measured by the total distance moved in the open field arena. B) MiR-mimic treatment does not affect anxiety-like behavior, as measured by the amount of time spent in the centre and corners of the open field arena. C) MiR-mimic treated groups do not differentiate better between novel and familiar arm than the control-treated group in the T-maze. D) After 10 days of training, both control and miR-mix-treated mice spend significantly more time in the target quadrant of the Morris Water Maze (one-sample t-test against 25 s, **, *p* < 0.01). E) Although both treatment groups spent significantly more time with the stranger mouse (Str1) than close to the empty cage in the Social Preference/Social Novelty test (one-sample t-test against 50%, **, *p* < 0.01), both do not differentiate between the novel (StrNov) and the familiar stranger (StrFam). F) Both control and miR-mix-treated mice spend significantly more time with the novel than the familiar object (one-sample t-test against 0, *, *p* < 0.05, **, *p* < 0.01). (PNG 3027 kb)

## Abbreviations

10 M: 10 months of age
4 M: 4 months of age
AD: Alzheimer’s disease
APPtg: APP^swe^/PS1^L166P^
Aβ: amyloid-β
CFR: Contextual fear response
Cp: Crossing points
DE: Differential expression
FISH: Fluorescent in situ hybridization
GO: Gene ontology
ISH: In situ hybridization
LFC: Log2-fold change
miRNA: microRNA
MWM: Morris water maze
NFT: Neurofibrillary tangle
NOR: Novel object recognition test
padj: Adjusted *p*-value after false discovery rate correction
RT: Room temperature
SPSN: Social preference/social novelty test
TAUtg: Thy-TAU22
Tg: Transgenic
Wt: Wild-type
