# Supplementary figures and images for: Deregulation of neuronal miRNAs induced by amyloid-β or TAU pathology
Source: Mol Neurodegener. 2018 Oct 12;13:54. doi: 10.1186/s13024-018-0285-1 (PMC6186090; doi:10.1186/s13024-018-0285-1)

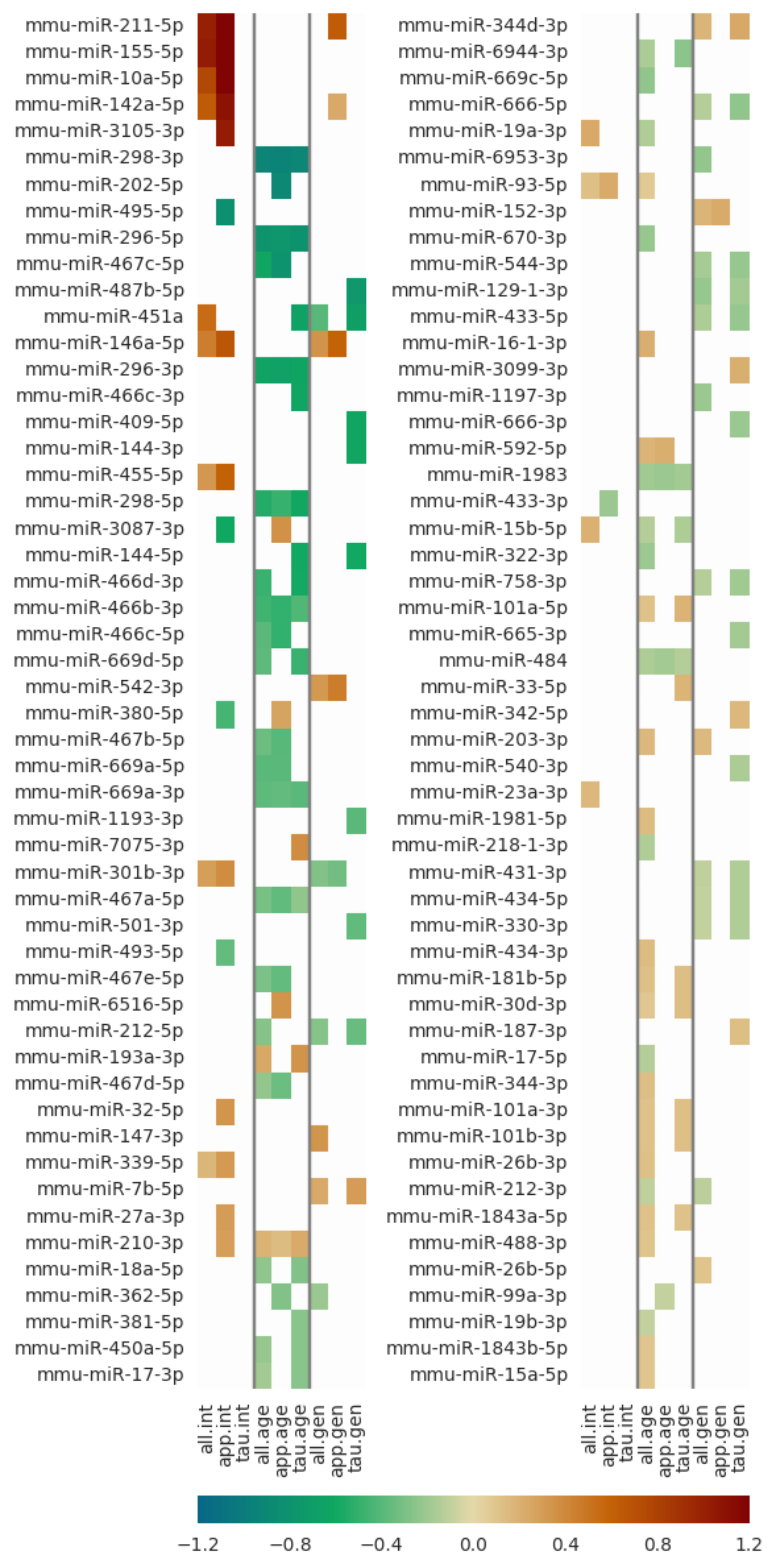

Supplement: Supplementary file 4 — Figure S3. Significantly differentially expressed miRNAs after statistical assessment of age*genotype interaction (int), age and genotype (gen) in APPtg and TAUtg mice combined (all) and separate. Colors represent log2(fold change) and only statistically significant (padj< 0.05) miRNAs are depicted. (PNG 2826 kb) [file 13024_2018_285_MOESM4_ESM.png]

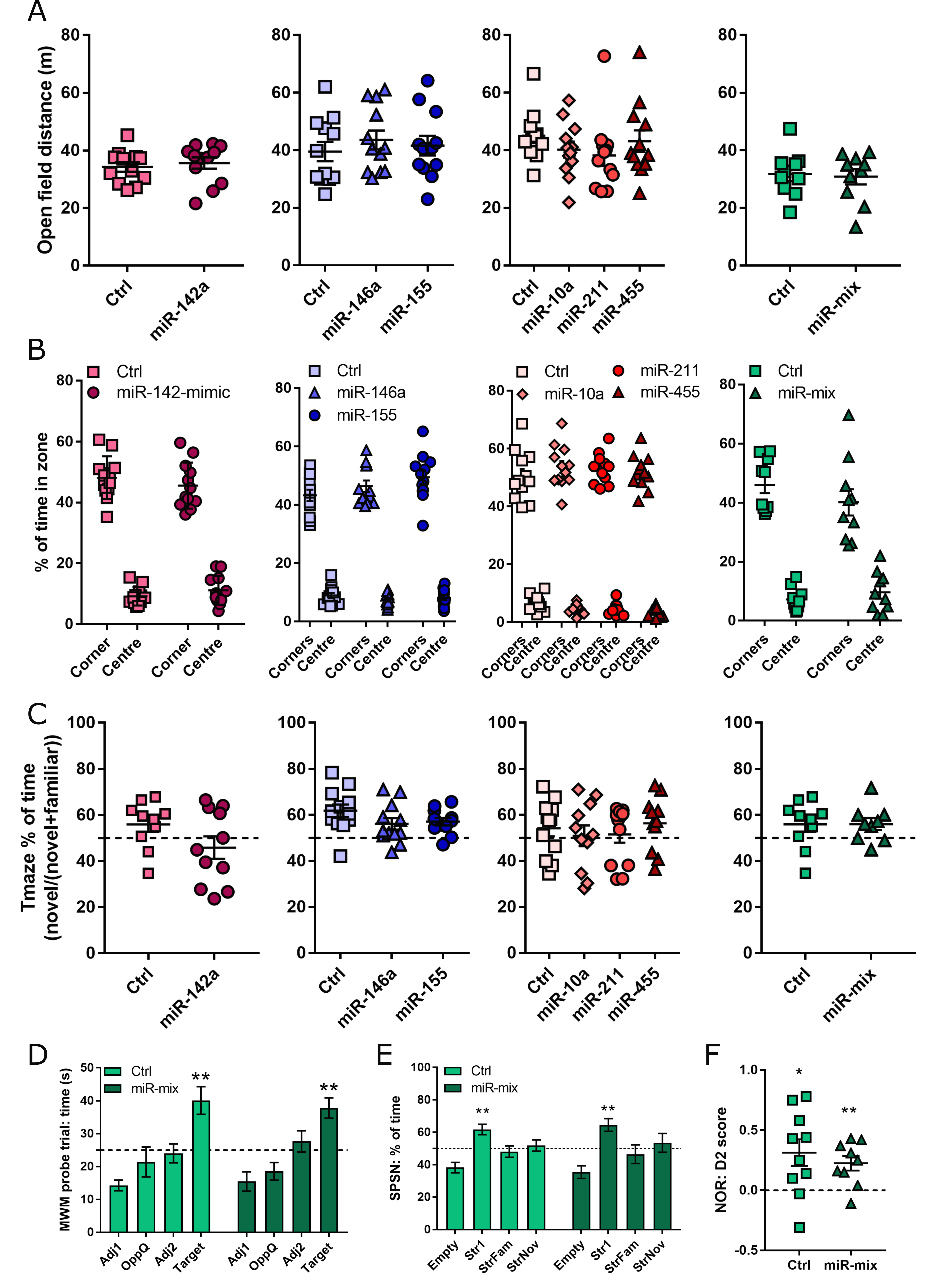

Supplement: Supplementary file 5 — Figure S4. Behavioral assessment after intracerebroventricular injections of individual or a mix of 6 miR-mimic oligos (miR-mix), or negative control (Ctrl). A) miRNA overexpression does not induce changes in locomotor behavior as measured by the total distance moved in the open field arena. B) MiR-mimic treatment does not affect anxiety-like behavior, as measured by the amount of time spent in the centre and corners of the open field arena. C) MiR-mimic treated groups do not differentiate better between novel and familiar arm than the control-treated group in the T-maze. D) After 10 days of training, both control and miR-mix-treated mice spend significantly more time in the target quadrant of the Morris Water Maze (one-sample t-test against 25 s, **, p < 0.01). E) Although both treatment groups spent significantly more time with the stranger mouse (Str1) than close to the empty cage in the Social Preference/Social Novelty test (one-sample t-test against 50%, **, p < 0.01), both do not differentiate between the novel (StrNov) and the familiar stranger (StrFam). F) Both control and miR-mix-treated mice spend significantly more time with the novel than the familiar object (one-sample t-test against 0, *, p < 0.05, **, p < 0.01). (PNG 3027 kb) [file 13024_2018_285_MOESM5_ESM.png]
